# Human Papillomavirus Type16- L1 VLP Production in Insect Cells

**Published:** 2013-08

**Authors:** Asghar Abdoli, Hoorieh Soleimanjahi, Fatemeh Fotouhi, Ali Teimoori, Shahram Pour Beiranvand, Zahra Kianmehr

**Affiliations:** 1 Department of Virology, Faculty of Medical Sciences, Tarbiat Modares University, Tehran, Iran; 2 Department of Anatomy, Faculty of Medical Sciences, Tarbiat Modares University, Tehran, Iran; 3 Influenza Research Lab, Department of Virology, Pasteur Institute of Iran, Tehran, Iran

**Keywords:** Baculovirus, Cervical cancer, HPV16- L1 VLP

## Abstract

***Objective(s):*** Infection by high-risk papillomavirus is regarded as the major risk factor in the development of cervical cancer. Recombinant DNA technology allows expression of the L1 major capsid protein of HPV in different expression systems, which has intrinsic capacity to self-assemble into viral-like particles (VLP). VLPS are non-infectious, highly immunogenic and can elicit neutralizing antibodies. VLP-based HPV vaccines can prevent persistent HPV infections and cervical cancer. In this study recombinant HPV-16 L1 protein was produced in Sf9 insect cells and VLP formation was confirmed.

***Materials and Methods: ***Complete *HPV-16 L1* gene was inserted into pFast HTa plasmid and transformed into *DH10BAC Escherichia coli *containing bacmid and helper plasmid. The recombinant Bacmid colonies turned to white and non-recombinant colonies harboring* L1* gene remained blue in the presence of X-gal and IPTG in colony selection strategy. To confirm the recombinant bacmid production, PCR was applied using specific L1 primers. To produce recombinant baculovirus, the recombinant bacmid DNA was extracted and transfected into Sf9 cells using Cellfectin. The expression of L1 in Sf9 cells was identified through SDS-PAGE and western blot analysis using specific L1 monoclonal antibody. Self-assembled HPV16L-VLPs in Sf9 cells was confirmed by electron microscopy.

***Results:*** The recombinant protein L1 was predominantly ~60 KD in SDS-PAGE with distinct immunoreactivity in western blot analysis and formed VLPS as confirmed by electron microscopy.

***Conclusion:*** Application of recombinant baculovirus containing *HPV-16 L1* gene will certainly prove to be a constructive tool in production of VLPs for prophylactic vaccine development as well as diagnostic tests.

## Introduction

Cervical cancer is one of the major health problems in women and the second most common cancer among them, with approximately 500,000 cases diagnosed resulting in 274,000 deaths worldwide every year (1). Clinical, molecular and epidemiological investigations have identified human papillomavirus (HPV) as the major cause of cervical cancer and cervical dysplasia. However, it is well known that most women have non-symptomatic HPV infections during their life (2). HPV is a non-enveloped, double-stranded DNA virus and over 100 different types of HPV have been identified (3, 4). High-risk HPV types that are commonly transmitted sexually are responsible for 99.7% of cervical cancer cases. Among the high-risk strains, HPV16 is the most closely associated with cervical carcinoma and detected in 50–60% of cervical cancers (5, 6). Therefore, HPV16- L1 is now the most significant target in production of preventive HPV vaccines. The HPV16 capsid is composed of major (L1) and minor (L2) proteins, and built up from 72 pentameric capsomers of L1 protein (55-60KD) arranged in an icosahedral array (7, 8). The discovery that the expression of the HPV16-L1 major capsid protein in different expression systems (e.g. insect cells or yeast) leads to formation of self-assembled virus-like particles (VLPs), initiated several studies. HPV16-VLPs resemble the native virions in size (55 nm) and shape, although, they do not carry the viral genome. They are highly immunogenic as they can induce high titers of neutralizing antibodies which have been shown to successfully prevent persistent HPV infections (-).

Recently, two prophylactic vaccines based on VLPs, Gardasil (Merck Sharpe & Dohme), and Cervarix (GlaxoSmithKline, GSK), have been introduced to the market (11, 12). Although these vaccines are highly efficient, the major drawbacks of these vaccines are the high production and purification costs due to their expression in eukaryotic cells (insect cells or yeast), besides they need cold chain and sterile needles for the intramuscular administration. Furthermore, three doses of vaccines are necessary to obtain efficient immune responses. Subsequently, they will likely remain unaffordable for developing countries, where, 83% of all cervical cancer cases occur (1, 13). An economically advantageous alternative to use of these vaccines might be the development technology production of HPV-VLPs based vaccines in our country.

In the previous study, we extracted* HPV16*-*L1* gene from paraffin embedded infected cervical tissues (14). In the present study we used the Bac to Bac baculovirus expression system to produce HPV16-L1 VLPs in Sf9 insect cells. 

## Material and Method

***Construction of recombinant pFastBacHTa***

The *HPV16*-L1 full genome was amplified from paraffin embedded tissues, cloned to pTZ57R/T vector (Fermentase, Lithuania) and confirmed by sequencing as described previously (14). The 1595 bp L1 ORF was excised from the cloning vector with *SalI* and *XhoI*(Ferments, Lithuani) and subcloned into the pFastBacHTa(pFB) donor plasmid of baculovirus expression system (Invitrogen, USA), which enabled the expression of a fusion protein with a N-terminus 6xHis-tag. The tranformants were verified with restriction enzymes analysis by *BamHI*. Preparation of competent cells, transformation of bacteria, DNA preparation, and electrophoresis in agarose gels were performed according to standard protocols (15).

***Generation of recombinant bacmid***

The recombinant donor plasmid pFB-HPV16 L1 was transformed into the *Escherichia coli* DH10Bac competent cells which contained the bacmid with a mini-attTn7 target site and the helper plasmid for site-specific transposition of the *HPV16*-L1 gene from the donor vector to a bacmid DNA through lacZ gene disruption. The transformed cells were plated onto the LB agar containing kanamycin (50 µg/mL), gentamicin (7 µg/mL), tetracycline (10 µg/mL), X-gal (100 µg/mL) and isopropylthio-β-galactoside (IPTG, 40 µg/mL) and incubated at 37 °C for 48 hr. The resultant recombinant bacmid produced white colonies in the presence of IPTG and X-gal, while non-recombinant bacmid remained blue. The high molecular weight bacmid DNA was isolated from the overnight cultures by alkaline lysis purification according to the instructions supplied by the manufacturer’s manual of Bac to Bac baculovirus expression system (Invitrogen, USA). Successful transposition was verified by PCR analysis using L1-specific primers (14).

***Transfection of Sf9 cells with recombinant bacmid DNA***

*Spodoptra frugipedra *(Sf9) cells were purchased from National Cell Bank (Pasteur Institute of Iran) and cultured at 27°C in Grace’s insect cell culture medium (GIBCO, Invitrogen, Germany), supplemented with 10% heat-inactivated fetal bovine serum (GIBCO, Invitrogen, Germany), 50 u/ml penicillin and 50 µg/ml streptomycin. Sf9 cells were transfected with isolated recombinant bacmid DNA using Cellfectin, a cationic lipid for production of the baculovirus particles according to the manufacturer’s instructions. Briefly, for each transfection, 9 10^5^ cells per well were seeded in a 6-well plate and allowed to attach for 1 hr. The bacmid DNA (1 µg of recombinant HPV16-L1 bacmid DNA) and Cellfectin (6 µl of reagent) were diluted separately in 100 µl of unsupplemented Grace’s medium without antibiotics, then mixed and incubated for 30 min at room temperature(RT) to form lipid-DNA complexes. The cells were washed with fresh medium, and incubated with lipid-DNA complex at 27°C for 5 hr. The transfection solution was removed and 2 ml supplemented Grace’s medium were added. Transfected Sf9 cells were incubated at 27°C for 72 hr for baculovirus production. Recombinant baculovirus production was monitored daily by visualization of the cytopathic effects (CPE) (16, 17).

For amplification of the baculovirus master stock, Sf9 cells were inoculated with proper amount of viral stock (corresponding to a MOI of 0.01-0.5) and incubated at 27°C for 48 hr. The culture medium was collected, clarified and titrated as plaque forming unit. For protein production, the cells were inoculated with recombinant baculovirus at a MOI of 10 and incubated at 27°C for 72 hr (18).

***Confirmation of HPV16L1 protein expression in Sf9 cells***

SDS-PAGE electrophoresis and western blot analysis were applied for verification of protein expression. The transfected Sf9 cells were harvested at 72 hr post infection (pi), the cell pellet was collected, washed three times with cold phosphate-buffered saline (PBS), resuspended in cell lysis buffer (50 mM Tris-HCl, pH 8.5, 5 mM 2-mercaptoethanol, 1 mM phenylmethylsulfonyl fluoride, 100mM KCl) and sonicated three times for 10 sec at 3 min intervals, with 50% power of the ultrasonicator. SDS-PAGE was performed in 12.5% acrylamide gel. The separated proteins were stained with 0.25% Coomassie blue or transferred to a nitrocellulose membrane. The membrane was blocked with Tris-buffered saline (TBS) containing 2% BSA for 1.5 hr (RT), washed and reacted with 1:100 dilution of anti-HPV16-L1 monoclonal antibody (abcam, USA) overnight (RT). The immunoreactive bands were visualized by the horseradish peroxidase (HRP) conjugated anti-mouse antibody (Biogen, Iran) for 2 hr (RT) and developed with 3, 3', 5, 5' tetra methyl benzidine (TMB) as substrate.

***Identification of virus like particles (VLPs) by transmission electron microscopy***

VLP formation was verified by electron microsco-

py (19). Briefly, Sf9 cell extracts were fixed in 2% paraformaldehyde and 0.1% glutaraldehyde in PBS. The cells were washed in Sabatini's solution and post-fixed with 1% osmium tetroxide. The samples were then passed through a graded alcohol series, infiltrated with propylene oxide and embedded in Epon 812. Ultrathin (60 nm) sections were cut with a diamond knife on an UC6 ultramicrotome (Leica), stained with uranyl acetate and lead citrate, mounted on 200-mesh grids and examined with a electron microscope( Zeiss EM 900 80KV) in Tarbiat Modares University, Faculty of Medical Sciences, Iran.

## Results

***Construction of recombinant pFastBacHTa***

**Figure 1:**
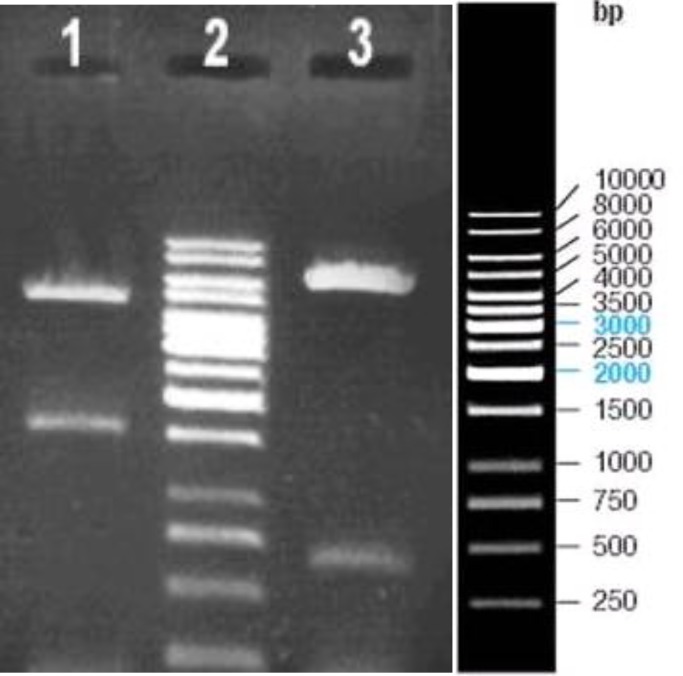
The confirmation of recombinant pFast HTa-L1 by restriction enzyme analysis: Lane 1; digestion of recombinant pFast HTa-L1 by *Sal*I and *Xho*I (4800 and 1595bp fragments), Lane 2; DNA size marker, Lane 3; digestion result of recombinant pFast HTa-L1 by *BamH*I (5735 and 660bp fragments

**Figure 2:**
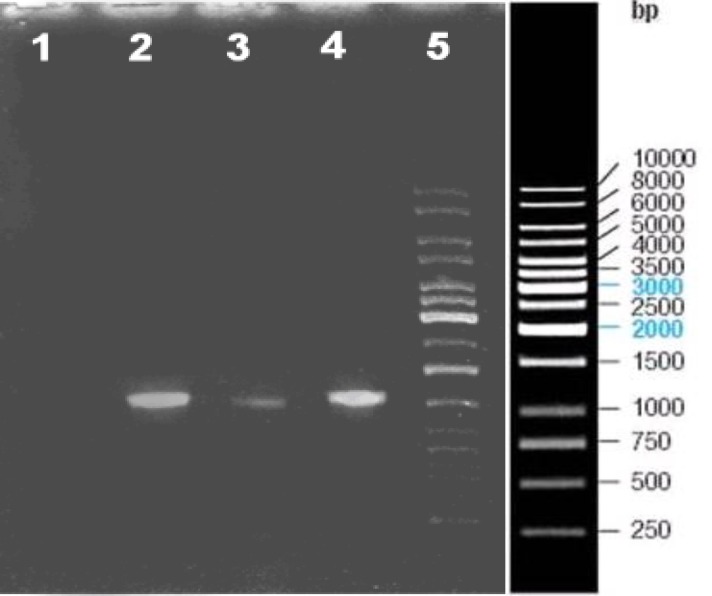
PCR analysis of the recombinant bacmid Lane 1; negative control, Lane 2; positive control. PCR of HPV-16 genome using specific L1 primers, Lane 3 and 4; insertion result of HPV16-L1 gene into bacmid DNA amplified with specific L1 primers, Lane 5; DNA size marker

The *HPV16*-L1 full genome was subcloned into the pFastHTa donor plasmid and the reco in the insert gene and the other in the vector which produces 5735 bp and 660 bp fragments as expected. The results have been provided in Figure 1. Since digestion and electrophoresis of high molecular DNA is not convenient, recombinant bacmid DNA containing *HPV16*-L1 was confirmed by PCR using L1 specific primers as demonstrated in Figure 2. 

***Generation of HPV16L1 bacmid DNA***

The recombinant plasmid pFB-HPV16L1 was successfully transformed into DH10Bac competent cells, and the recombinant bacmid DNA isolated from the transformed DH10Bac cells was used for Sf9 cell transfection. After culturing for 48 hr, the cells became rounded, swelling, and more refractive, and gradually detached from the flask, whereas the growth of non-transfected cells was normal.

***Expression and identification of HPV16-L1 protein***

**Figure 3:**
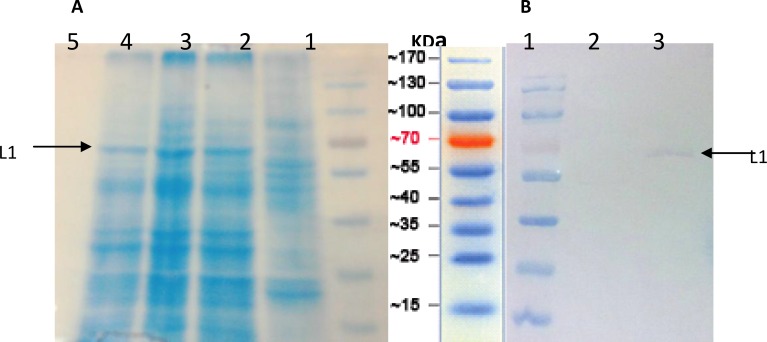
SDS-PAGE and Western blot analysis of HPV16- L1 protein expressed in Sf9 cells. (A) SDS-PAGE.. Lane 1; Protein Marker, Lane 2; uninfected Sf9 cells, and Lane 3, 4 and 5; Sf9 cells transfected with recombinant HPV16-L1 gene bacmid DNA. (B) Western blot analysis using the anti-HPV16-L1 monoclonal antibody. Lane 1; Protein marker, Lane 2; uninfected Sf9 cells, and Lane 3; Sf9 cells transfected with recombinant HPV16-L1 gene bacmid DNA. As shown un-specific bands didn’t observed using monoclonal antibody

Protein extracted from transfected Sf9 cells was a distinct band of ~60 KD as shown by SDS-PAGE [Figure 3(A)], which is consistent with the known molecular weight of HPV16 L1. It was capable of reacting with anti-HPV16L1 monoclonal antibody [Figure 3(B)]. 

***Identification of virus like particles (VLPs) by transmission electron microscopy***

**Figure 4:**
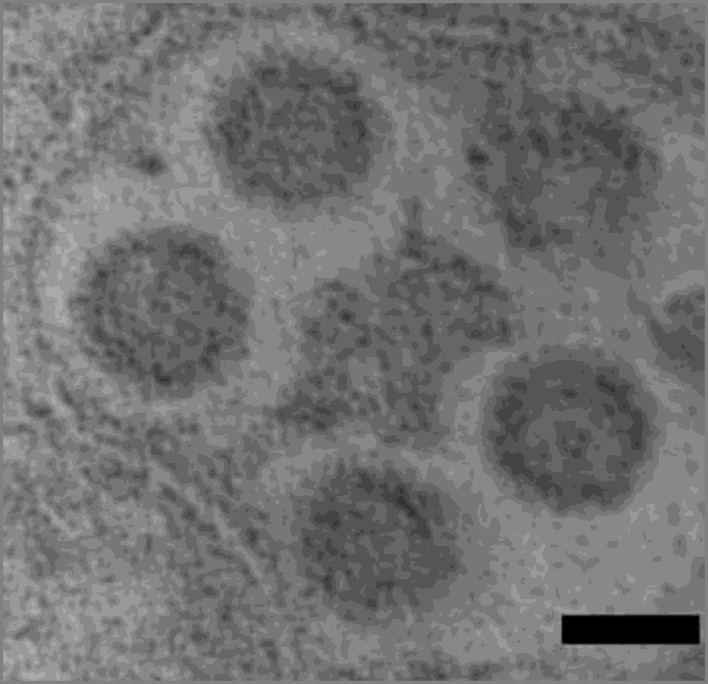
Self assembled HPV16 L1 VLPs stained with uranyl acetate and lead citrate analyzed under an electron microscope. Bar 50 nm

The VLPs were identified from insect cells infected with recombinant baculovirus expressing *HPV16*-L1 gene. Transmission electron microscopy demonstrat- ed the appropriate morphology of the self assembled recombinant HPV16L1-VLPs (50 nm), which was indicated in Figure 4.

## Discussion

The expression of HPV16- L1 protein in eukaryotic cells results in the formation of VLPs that mimic the natural virus structure and elicit high titers of virus-neutralizing antibodies in animal models and humans (19, 20). HPV16 L1-VLPs have been widely accepted as the best candidate of HPV16 prophylactic vaccines, and FDA has approved that the HPV16 VLP vaccine could be used for clinical trial (10).

High prevalence of HPV 16 has been reported in our country, therefore, newly designed vaccines should consist of high risk papillomaviruses specially type 16 (-). In this study, we are going to start to develop the production technology of HPV16-L1VLP based vaccines. A variety of eukaryotic expression systems are available for expression of recombinant proteins. Recombinant baculovirus which can grow and propagate in insect cell lines or larvae of several insect species were chosen for safety issues and capability of baculovirus genome for accommodating large exogenous genes. Furthermore, recombinant baculovirus is feasible for further processing, modification, targeting to appropriate cellular destinations and acting as authentic counterparts (22) .

The recombinant bacmid containing HPV16- L1 gene was transfected into Sf9 cells. SDS-PAGE and immunobloting analysis showed expression of ~60KD recombinant L1 proteins, which is in agreement with the other published reports (9, 24). Electron micrographs demonstrated the naive morphology of the self assembled recombinant VLPs in Sf9 cells. Our results which are consistent with other studies indicate that L1, the major capsid protein of HPVs, has the intrinsic capacity to self assemble into VLPs in the absence of L2 or other papillomavirus proteins (-).

## Conclusion

In summary, this study describes the successful expression of human Papilloma virus type16- L1 gene in insect cells. The HPV16- L1 protein is capable of independently assembling into virus-like particles (VLPs). HPV16- L1 VLPs produced in the baculovirus system can be considered potential candidates for prophylactic vaccine development and may be useful in seroepidemiological study of HPV16. It may also play a role in the identification and characterization of HPV capsid formation and HPV cell receptors. In a future study, we are going to formulate HPV16-L1VLPs with proper adjuvant and evaluate its efficiency as a vaccine candidate in mice model. 
